# Supplementary material for: Content and Mechanism of Action of National Antimicrobial Stewardship Interventions on Management of Respiratory Tract Infections in Primary and Community Care
Source: Antibiotics (Basel). 2020 Aug 13;9(8):512. doi: 10.3390/antibiotics9080512 (PMC7460499; doi:10.3390/antibiotics9080512)
Supplement: Supplementary file 1 [file antibiotics-09-00512-s001.pdf]

antibiotics

MDPI

Article

Content and Mechanism of Action of National Antimicrobial Stewardship Interventions on Management of Respiratory Tract Infections in Primary and Community Care

Lou Atkins [1,*], Tim Chadborn [2], Paulina Bondaronek [2], Diane Ashiru-Oredope [2], Elizabeth Beech [3], Natalie Herd [1], Victoria de La Morinière [4], Marta González-Iraizoz [2], Susan Hopkins [2], Cliodna McNulty [5] and Anna Sallis [2]

1. Center for Behavior Change, University College London, Alexandra House, 7–19 Queens Square, WC1N 3AZ London, UK; natalie.herd@gmail.com
2. Public Health England Behavioral Insights (PHEBI), Public Health England, Wellington House, 133–155 Waterloo Road, SE1 8UG London, UK; tim.chadborn@phe.gov.uk (T.C.); paulina.bondaronek@phe.gov.uk (P.B.); diane.ashiru-oredope@phe.gov.uk (D.A.-O.); M.Gonzalez-Iraizoz@warwick.ac.uk (M.G.-I.); susan.hopkins@phe.gov.uk (S.H.); anna.sallis@phe.gov.uk (A.S.)
3. NHS England and NHS Improvement, Wellington House, 1st Floor, 133-155 Waterloo Road, SE1 8UG London, UK; elizabeth.beech@nhs.net
4. Chelsea and Westminster Hospital NHS Foundation Trust, SW10 9NH London, UK; Victoria.delamoriniere@chelwest.nhs.uk
5. Public Health England, Primary Care Unit, Twyver House, Bruton Way, GL1 1DQ Gloucester, UK; Cliodna.McNulty@phe.gov.uk

* Correspondence: l.atkins@ucl.ac.uk

Received: 22 June 2020; Accepted: 10 August 2020; Published: date

Table S1. Labels, definitions, and examples of COM-B and Theoretical Domains Framework.

COM-B model.

**COM-B model component**<br>**Definition**	***Example***
**Physical capability**<br>Physical skill, strength, or stamina	*Having the skill to take a blood sample*
**Psychological capability**<br>Knowledge or psychological skills, strength, or stamina to engage in the necessary mental processes	*Understanding the impact of CO² on the environment*
**Physical opportunity**<br>Opportunity afforded by the environment involving time, resources, locations, cues, physical 'affordance'	*Being able to go running because one owns appropriate shoes*

**Social opportunity**<br>Opportunity afforded by interpersonal influences, social cues and cultural norms that influence the way that we think about things, e.g., the words and concepts that make up our language	*Being able to smoke in the house of someone who smokes but not in the middle of a boardroom meeting*
**Reflective motivation**<br>Reflective processes involving plans (self-conscious intentions) and evaluations (beliefs about what is good and bad)	*Intending to stop smoking*
**Automatic motivation**<br>Automatic processes involving emotional reactions, desires (wants and needs), impulses, inhibitions, drive states, and reflex responses	*Feeling anticipated pleasure at the prospect of eating a piece of chocolate cake*

Table S2. Behavior Change Wheel labels, definitions, and examples.

**Intervention Types**		
**Intervention Type**	**Definition**	***Example Of Intervention Function***
**Education**	Increasing knowledge or understanding	*Providing information to promote healthy eating*
**Persuasion**	Using communication to induce positive or negative feelings or stimulate action	*Using imagery to motivate increases in physical activity*
**Incentivization**	Creating an expectation of reward	*Using prize draws to induce attempts to stop smoking*
**Coercion**	Creating an expectation of punishment or cost	*Raising the financial cost to reduce excessive alcohol consumption*
**Training**	Imparting skills	*Advanced driver training to increase safe driving*
**Restriction**	Using rules to reduce the opportunity to engage in the target behavior (or to increase the target behavior by reducing the opportunity to engage in competing behaviors)	*Prohibiting sales of solvents to people under 18 to reduce use for intoxication*
**Environmental restructuring**	Changing the physical or social context	*Providing on-screen prompts for GPs to ask about smoking behavior*
**Modeling**	Providing an example for people to aspire to or imitate	*Using TV drama scenes involving safe-sex practices to increase condom use*
**Enablement**	Increasing means/reducing barriers to increase capability (beyond education and training) or opportunity (beyond environmental restructuring)	*Behavioral support for smoking cessation, medication for cognitive deficits, surgery to reduce obesity, prostheses to promote physical activity*
**Policy Option Definitions.**		
**Policy options**	**Definition**	**Example**

**Communication/ marketing**	Using print, electronic, telephonic or broadcast media	*Conducting mass media campaigns*
**Guidelines**	Creating documents that recommend or mandate practice. This includes all changes to service provision	*Producing and disseminating treatment protocols*
**Fiscal measures**	Using the tax system to reduce or increase the financial cost	*Increasing duty or increasing anti-smuggling activities*
**Regulation**	Establishing rules or principles of behavior or practice	*Establishing voluntary agreements on advertising*
**Legislation**	Making or changing laws	*Prohibiting sale or use*
**Environmental/social planning**	Designing and/or controlling the physical or social environment	*Using town planning*
**Service provision**	Delivering a service	*Establishing support services in workplaces, communities etc.*

Table S3: Sources from which AMS RTI behaviors were identified.

**Behavior**	**Source**
**Patients/Public (and or carers)**	
Self-care and/or obtain pharmacy advice for signs and symptoms of self-limiting respiratory tract infections prior to, or instead of, a primary care consultation.	NICE guidance [NG63], p.7
	NICE guidance [NG79], p.10
Do not request antibiotics at primary care consultations for symptoms of self-limiting RTIs.	PHE (2017) Antibiotic awareness: important messages on antibiotic use
Use back-up prescriptions as directed by a suitably qualified healthcare professional (HCP).	NICE guidance [NG63], p.14
Take antibiotics as prescribed (do not save for later use or share with others) by a suitably qualified HCP.	NICE guidance [NG63], p.8
	PHE (2017) Antibiotic Awareness Key Messages Key Messages, p.8:
Return unwanted antibiotics to the pharmacy.	NICE guidance [NG63], p.8:
**Primary care prescribers (including non-medical prescribers, such as nurses and pharmacists)**	
Follow/adhere to local antibiotic formulary—general behaviors	
Prescribe an antibiotic only when there is likely to be clear clinical benefit, (using fever PAIN or CENTOR for sore throat). OR Do not issue an immediate prescription for an antimicrobial to a patient who is likely to have a self-limiting condition.	NICE guidance [NG84], p. 24
	NICE guideline [NG15], p. 15:

	PHE (2017) Management and treatment of common infections
	Antibiotic guidance for primary care: For consultation and local adaptation, p.5
	PHE (2017) Antibiotic Awareness Key Messages Key Messages, p.12.
Give alternative, non-antibiotic self-care advice, where appropriate.	NICE guidance [NG63], p.13
	NICE guideline [NG15], p. 15
Use/share written self-care resources/leaflets when issuing self-care advice for symptoms of self-limiting RTIs.	NICE guidance [NG63], p.13
	PHE (2017) Antibiotic Awareness Key Messages Key Messages, p.6
Provide safety netting advice whether or not the patient has been prescribed antibiotics (e.g., what to do if condition gets worse or side effects of medication).	NICE guidance [NG63], p.14
When an antibiotic is indicated prescribe the narrowest spectrum antibiotic possible, for the right duration, at the right dose.	Infection prevention and control (QS61), p. 13
	NICE guidance [NG84], p.20
Provide 'delayed/back-up' antibiotic strategy where appropriate	NICE guidance [NG79], p.18
	PHE (2017) Management and treatment of common infections
	PHE (2017) Antibiotic Awareness Key Messages: important messages on antibiotic use p.14
Explain the prescribing decision to the patient, including where appropriate, the benefits and harms of antibiotics.	Quality standard [QS121]: Antimicrobial stewardship, p.14
Document, in patients records, clinical diagnosis (including symptoms) if prescribing an immediate or back up antimicrobial and/or giving self-care advice.	NICE guideline [NG15], p. 14
	Quality standard [QS121], p.20
Undertake POCT in patients 18–65 years old presenting with acute cough/bronchitis in whom antibiotics are being considered.	PHE (2017) Management and treatment of common infections, p.6

	Pneumonia in adults: diagnosis and management (CG191), p 8
Limit prescribing over the telephone to exceptional cases for self-limiting RTIs	PHE (2017) Management and treatment of common infections, p.5
**Community pharmacists and pharmacy staff**	
Provide self-care advice for patients with symptoms of self-limiting RTIs, instead of, following or prior to referral to a primary care clinician, giving safety netting advice where appropriate.	NICE guidance [NG63], p. 13
	NICE guidance [NG63], p. 15
Use/share written resources with the public when providing self-care advice for self-limiting RTIs.	NICE guidance [NG63], p. 13; p.63
When giving an antibiotic prescription for a self-limiting RTI, inform the patients of the dose and duration or to take their antibiotics exactly as prescribed.	RPS (2017) Antimicrobial stewardship: A Quick reference guide, p. 2
Check that antibiotic prescriptions comply with local guidance and query with the prescriber for those that do not.	RPS (2017) Antimicrobial stewardship: A Quick reference guide, p. 5
Accept and dispose appropriately of returned antibiotics.	NHS Community Pharmacy Contractual Framework Essential Service
**Providers and commissioners**	
Provide education and training in prudent antimicrobial use/AMR (using the antimicrobial resistance and stewardship competencies as a framework).	NICE guideline [NG15], p.9
	NICE guideline [NG15] p.12
Commission, develop or implement interventions (e.g., guidance, services, programs, or campaigns) to support AMS/tackle AMR	NICE guidance [NG15], p.10
	Quality standard [QS61], p.11
	Infection prevention and control (QS61),p.11:
Commission, develop or implement interventions (e.g., guidance, services, programs, or campaigns) to support self-care.	NICE guidance [NG63], p. 33
Monitor antibiotic prescribing in relation to local and national resistance patterns or targets.	NICE guideline [NG15] p.9
	NICE guideline [NG15], p.12

	NICE support for commissioning for infection prevention and control (2014), p. 8
	Infection prevention and control (QS61), p15
	Quality standard [QS121]
Promote current national guidelines, or promote/develop local guidelines on antimicrobial prescribing among all prescribers, providing updates if the guidelines change.	NICE guideline [NG15], p.10
	NICE guideline [NG15], p.13
Provide regular feedback on antimicrobial prescribing and resistance indicators at prescriber, team and organization level benchmarked against local or national antimicrobial prescribing/resistance rates.	NICE guidance [NG15], p. 9, 10
	Quality standard [QS121]
Provide feedback to prescribers on patient safety incidents related to antimicrobial use, including hospital admissions for potentially avoidable life-threatening infections, infections with Clostridium difficile or adverse drug reactions, such as anaphylaxis.	NICE guideline [NG15]
Providers have a formulary in place for antibiotic prescribing *	Quality standard [QS61], p.10
	Quality standard [QS61], p.11
	NICE guidance [NG15], p. 10,11
Commissioners seek evidence/providers make evidence available for adherence to local or national guidance for antibiotic prescribing.**	Quality standard [QS121]
Commissioners ensure information and resources are available for healthcare professionals to use during consultations with people seeking advice about managing self-limiting RTIs. **	NICE guidance [NG63], p.7
Reduce antibiotic prescribing/antimicrobial resistance—general behaviors.	

* Providers only ** Commissioners only.

Table S4. Summary of intervention content, mechanism of action and target behavior

	Behaviour (Number Of Behaviours)	Number Of Interventions Targeting The Behavior	Intervention Names	COM-B	TDF	Intervention Types	Bcts	Policy Options
	**Patients/Public (and or carers) (n=5)**							
1	Self-care and/or obtain pharmacy advice for signs and symptoms of self-limiting respiratory tract infections prior to, or instead of, a primary care consultation.	13	Public Health England Antibiotic Guardian; TARGET Antibiotics Toolkit; Treat Yourself Better; NHS website advice on common cold; NICE Respiratory tract infections (self-limiting): prescribing antibiotics [CG69]; Self Care Forum: Factsheet 7 (Cough in Adults); Factsheet 12 (Common Cold); Public Health England Keep Antibiotics Working campaign; Department of Health & Social Care 'Take Care not Antibiotics' videos; Patient.info webpages on colds, sore throats, antibiotics, bronchitis and sinusitis; Self Care Forum: Self Care Week; Public Health England 'Beat the Bugs' course; British Society for Antimicrobial Chemotherapy: Antibiotic Action.	Psychological capability<br>Physical capability<br>Reflective motivation<br>Automatic motivation<br>Physical opportunity<br>Social opportunity	Knowledge<br>Skills<br>Memory, attention and decision processes<br>Beliefs about consequences<br>Intention<br>Social professional role and identity<br>Reinforcement<br>Environmental context and resources<br>Social influences	Education<br>Persuasion<br>Incentivization<br>Training<br>Enablement	Commitment<br>Instruction on how to perform the behavior<br>Information about health consequences<br>Credible source<br>Identification of self as role model<br>Social support practical<br>Prompts/cues<br>Pharmacological support<br>Demonstration of the behavior<br>Adding objects to the environment<br>Social comparison<br>Information about social and environmental consequences<br>Behavioral practice/rehearsal<br>Non-specific reward	Communication/ marketing<br>Guidelines<br>Service provision
2	Do not request antibiotics at primary care consultations for symptoms of self-limiting RTIs.	12	Public Health England Antibiotic Guardian; NICE Infection Prevention and Control [QS61]; TARGET Antibiotics Toolkit; NHS website advice on common cold; Self Care Forum: Factsheet 7 (Cough in Adults); Factsheet 12 (Common Cold); Public Health England Keep Antibiotics Working campaign; NICE Sinusitis (acute): antimicrobial prescribing [NG79]; Patient.info webpages on colds, sore throats, antibiotics, bronchitis and sinusitis; Health Education England 'Awareness of Antimicrobial Resistance (AMR) Animation'; Self Care Forum: Self Care Week; Public Health England 'Beat the Bugs' course; British Society for Antimicrobial Chemotherapy: Antibiotic Action.	Psychological capability<br>Reflective motivation<br>Automatic motivation<br>Social opportunity	Knowledge<br>Memory, attention, decision making<br>Beliefs about consequences<br>Intention<br>Emotion<br>Social influences<br>Social professional role and identity<br>Skills<br>Behavioral regulation	Training<br>Enablement<br>Education<br>Persuasion<br>Modeling	Instruction on how to perform the behavior<br>Prompts/cues<br>Information about health consequences<br>Salience of consequences<br>Credible source<br>Demonstration of the behavior<br>Identification of self as role model<br>Action planning<br>Behavioral practice/rehearsal	Guidelines<br>Communication/ marketing<br>Service provision
3	Use back-up prescriptions as directed by a suitably qualified healthcare professional (HCP).	3	TARGET Antibiotics Toolkit; NICE Sinusitis (acute): antimicrobial prescribing [NG79]; Patient.info webpages on colds, sore throats, antibiotics, bronchitis and sinusitis.	Psychological capability	Knowledge<br>Behavioral regulation	Enablement<br>Training<br>Education	Instruction on how to perform the behavior<br>Information about health consequences<br>Action-planning	Guidelines<br>Communication/ marketing<br>Service provision

4	Take antibiotics as prescribed (do not save for later use or share with others) by a suitably qualified HCP.	4	Public Health England Antibiotic Guardian; TARGET Antibiotics Toolkit; Patient.info webpages on colds, sore throats, antibiotics, bronchitis and sinusitis; Public Health England 'Beat the Bugs' course.	Reflective motivation<br>Psychological capability	Intention<br>Knowledge<br>Skills<br>Beliefs about consequences	Enablement<br>Training<br>Education<br>Persuasion	Commitment<br>Instruction on how to perform the behavior<br>Information about health consequences	Service provision<br>Communication/ marketing
5	Return unwanted antibiotics to the pharmacy.	3	Public Health England Antibiotic Guardian; TARGET Antibiotics Toolkit; Public Health England 'Beat the Bugs' course.	Reflective motivation<br>Psychological capability	Intention<br>Skills	Enablement<br>Training	Commitment<br>Instruction on how to perform the behavior	Service provision<br>Communication/ marketing
	**Primary care prescribers (including non-medical prescribers such as nurses and pharmacists) n=11**							
6	Follow/adhere to local antibiotic formulary - general behaviors.	13	UK Department of Health and Public Health England Antimicrobial Prescribing and Stewardship Competencies; NICE Antimicrobial stewardship: systems and processes for effective antimicrobial medicine use [NG15]; NICE Infection Prevention and Control [QS61]; TARGET Antibiotics Toolkit; UK Five Year Antimicrobial Resistance Strategy 2013 to 2018; Center for Pharmacy Postgraduate Education distance course: Antibacterial resistance - a global threat to public health: the role of the pharmacy team; UK Clinical Pharmacy Association / Royal Pharmaceutical Society – professional practice curriculum; Public health England Managing Common Infections Guidance; NICE Antimicrobial Stewardship [QS121]; Stemming the Tide of Antibiotic Resistance (STAR) e-learning; CENTOR; Health Education England 'Antimicrobial Resistance: A Guide for GPs'; NICE Sore throat (acute): antimicrobial prescribing [NG84].	Psychological capability<br>Physical opportunity<br>Reflective motivation<br>Social opportunity<br>Automatic motivation	Skills<br>Knowledge<br>Behavioral regulation<br>Environmental context and resources<br>Intention<br>Beliefs about consequences<br>Social influences<br>Reinforcement<br>Social professional role and identity<br>Memory, attention and decision making	Training<br>Education<br>Enablement<br>Persuasion<br>Modeling<br>Incentivization	Instruction on how to perform the behavior<br>Self-monitoring of the behavior<br>Adding objects to the environment<br>Credible source<br>Information about health consequences<br>Demonstration of the behavior<br>Non-specific reward<br>Identification of self as role model<br>Social support practical<br>Action planning	Guidelines<br>Service provision<br>Communication / Marketing
7	Prescribe an antibiotic only when there is likely to be clear clinical benefit, (using feverPAIN or CENTOR for sore throat). OR Do not issue an immediate prescription for an antimicrobial to a patient who is likely to have a self-limiting condition .	19	Public Health England Antibiotic Guardian; UK Department of Health and Public Health England Antimicrobial Prescribing and Stewardship Competencies; NICE Antimicrobial stewardship: systems and processes for effective antimicrobial medicine use [NG15]; NICE Infection Prevention and Control [QS61]; TARGET Antibiotics Toolkit; UK Chief Medical Officer letter to high prescribers of antibiotics; UK Five Year Antimicrobial Resistance Strategy 2013 to 2018; Center for Pharmacy Postgraduate Education distance course: Antibacterial resistance - a global threat to public health: the role of the pharmacy team; FeverPAIN; Public health England Managing Common Infections Guidance; NICE Respiratory tract infections (self-limiting): prescribing antibiotics [CG69]; NICE Antimicrobial Stewardship [QS121]; Managing Acute Respiratory Tract Infections (MARTI) e-learning; Stemming the Tide of Antibiotic Resistance (STAR) e-learning; CENTOR; Health Education England 'Antimicrobial Resistance: A Guide for GPs'; NICE Sinusitis (acute): antimicrobial prescribing [NG79]; NICE Sore throat (acute): antimicrobial prescribing [NG84]; British Society for Antimicrobial Chemotherapy: Antibiotic Action.	Reflective motivation<br>Psychological capability<br>Automatic motivation<br>Physical opportunity	Social professional role and identity<br>Skills<br>Knowledge<br>Memory, attention and decision making<br>Reinforcement<br>Beliefs about consequences<br>Behavioral regulation<br>Optimism<br>Emotion<br>Intention<br>Environmental context and resources	Enablement<br>Training<br>Education<br>Incentivization<br>Persuasion<br>Coercion	Identification of self as role model<br>Instruction on how to perform the behavior<br>Information about health consequences<br>Action planning<br>Reward non-material<br>Social comparison<br>Credible source<br>Feedback on behavior<br>Future punishment<br>Adding objects to the environment<br>Behavioral practice/rehearsal<br>Non-specific reward<br>Self-monitoring of outcome(s) of behavior<br>Feedback on outcome(s) of the behavior<br>Information about social and environmental consequences<br>Salience of	Service provision<br>Guidelines<br>Communication/ marketing

							consequences<br>Demonstration of the behavior<br>Problem solving	
8	Give alternative, non-antibiotic self-care advice, where appropriate.	12	Public Health England Antibiotic Guardian; UK Department of Health and Public Health England Antimicrobial Prescribing and Stewardship Competencies; NICE Antimicrobial stewardship: systems and processes for effective antimicrobial medicine use [NG15]; TARGET Antibiotics Toolkit; UK Chief Medical Officer letter to high prescribers of antibiotics; NICE Antimicrobial stewardship: changing risk-related behaviors in the general population [NG63]; NICE Respiratory tract infections (self-limiting): prescribing antibiotics [CG69]; NICE Antimicrobial Stewardship [QS121]; Managing Acute Respiratory Tract Infections (MARTI) e-learning; Health Education England 'Antimicrobial Resistance: A Guide for GPs'; NICE Sinusitis (acute): antimicrobial prescribing [NG79]; NICE Sore throat (acute): antimicrobial prescribing [NG84].	Psychological capability<br>Reflective motivation<br>Automatic motivation	Knowledge<br>Skills<br>Memory, attention and decision processes<br>Beliefs about consequences<br>Intentions<br>Reinforcement	Education<br>Incentivization<br>Training<br>Enablement	Action planning<br>Commitment<br>Framing reframing<br>Information about health consequences<br>Information about social environmental consequences<br>Instruction on how to perform the behavior<br>Non-specific reward	Service provision<br>Guidelines<br>Communication/ marketing
9	Use/share written self-care resources/leaflets when issuing self-care advice for symptoms of self-limiting RTIs.	9	Public Health England Antibiotic Guardian; UK Department of Health and Public Health England Antimicrobial Prescribing and Stewardship Competencies; TARGET Antibiotics Toolkit; UK Chief Medical Officer letter to high prescribers of antibiotics; PrescQIPP Antimicrobial Stewardship; NICE Antimicrobial stewardship: changing risk-related behaviors in the general population [NG63]; Center for Pharmacy Postgraduate Education distance course: Antibacterial resistance - a global threat to public health: the role of the pharmacy team; Managing Acute Respiratory Tract Infections (MARTI) e-learning; Health Education England 'Antimicrobial Resistance: A Guide for GPs'.	Psychological capability<br>Physical opportunity<br>Reflective motivation	Knowledge<br>Skills<br>Behavioral regulation<br>Beliefs about consequences<br>Intentions<br>Environmental context and resources	Education<br>Persuasion<br>Training<br>Enablement	Adding objects to the environment<br>Behavioral substitution<br>Commitment<br>Credible source<br>Demonstration of the behavior<br>Information about health consequences<br>Instruction on how to perform the behavior<br>Self-monitoring of behavior<br>Social support (practical)	Service provision<br>Guidelines<br>Communication/ marketing
10	Provide safety netting advice whether or not the patient has been prescribed antibiotics (e.g., what to do if condition gets worse or side effects of medication).	7	Public Health England Antibiotic Guardian; NICE Antimicrobial stewardship: systems and processes for effective antimicrobial medicine use [NG15]; TARGET Antibiotics Toolkit; NICE Antimicrobial stewardship: changing risk-related behaviors in the general population [NG63]; Managing Acute Respiratory Tract Infections (MARTI) e-learning; NICE Sinusitis (acute): antimicrobial prescribing [NG79]; NICE Sore throat (acute): antimicrobial prescribing [NG84].	Psychological capability<br>Reflective motivation	Knowledge<br>Skills<br>Memory, attention and decision processes<br>Intentions	Training<br>Enablement	Action planning<br>Commitment<br>Instruction on how to perform the behavior	Guidelines<br>Service provision
11	When an antibiotic is indicated prescribe the narrowest spectrum antibiotic possible, for the right duration, at the right dose.	9	UK Department of Health and Public Health England Antimicrobial Prescribing and Stewardship Competencies; NICE Infection Prevention and Control [QS61]; TARGET Antibiotics Toolkit; UK Five Year Antimicrobial Resistance Strategy 2013 to 2018; Center for Pharmacy Postgraduate Education distance course: Antibacterial resistance - a global threat to public health: the role of the pharmacy team; Public health England Managing Common Infections Guidance; Managing Acute Respiratory Tract Infections (MARTI) e-learning; NICE Sinusitis (acute): antimicrobial prescribing [NG79]; NICE Sore throat (acute): antimicrobial prescribing [NG84].	Psychological capability<br>Reflective motivation	Knowledge<br>Skills<br>Behavioral regulation<br>Beliefs about consequences	Education<br>Training	Information about health consequences<br>Instruction on how to perform the behavior<br>Self-monitoring of outcomes of behavior<br>Social support (practical)	Guidelines<br>Service provision
12	Provide 'delayed/back-up' antibiotic strategy where appropriate.	14	Public Health England Antibiotic Guardian; UK Department of Health and Public Health England Antimicrobial Prescribing and Stewardship Competencies; NICE Antimicrobial stewardship: systems and processes for effective antimicrobial medicine use [NG15]; NICE Infection Prevention and Control [QS61]; TARGET Antibiotics Toolkit; UK Chief Medical Officer letter to high prescribers of antibiotics; UK Five Year Antimicrobial Resistance Strategy 2013 to 2018; Center for Pharmacy Postgraduate Education distance course: Antibacterial resistance - a global threat to public health: the role of the pharmacy team; Public health England Managing Common Infections Guidance; NICE Respiratory tract infections (self-limiting): prescribing antibiotics [CG69]; NICE Antimicrobial Stewardship [QS121]; Managing	Psychological capability<br>Reflective motivation	Behavioral regulation<br>Beliefs about consequences<br>Intention<br>Knowledge<br>Memory, attention and decision processes<br>Skills	Education<br>Enablement<br>Persuasion<br>Training	Action planning<br>Commitment<br>Credible source<br>Information about health consequences<br>Information about social environmental consequences<br>Instruction on how to perform the behavior	Service provision<br>Guidelines<br>Communication/ marketing

			Acute Respiratory Tract Infections (MARTI) e-learning; NICE Sinusitis (acute): antimicrobial prescribing [NG79]; NICE Sore throat (acute): antimicrobial prescribing [NG84].		Social/professional role and identity		Self-monitoring of behavior	
13	Explain the prescribing decision to the patient, including where appropriate, the benefits and harms of antibiotics.	10	UK Department of Health and Public Health England Antimicrobial Prescribing and Stewardship Competencies; NICE Antimicrobial stewardship: systems and processes for effective antimicrobial medicine use [NG15]; TARGET Antibiotics Toolkit; UK Five Year Antimicrobial Resistance Strategy 2013 to 2018; NICE Antimicrobial stewardship: changing risk-related behaviors in the general population [NG63]; NICE Antimicrobial Stewardship [QS121]; Managing Acute Respiratory Tract Infections (MARTI) e-learning; Stemming the Tide of Antibiotic Resistance (STAR) e-learning; Health Education England 'Antimicrobial Resistance: A Guide for GPs'; NICE Sore throat (acute): antimicrobial prescribing [NG84].	Psychological capability<br>Reflective motivation<br>Social opportunity	Behavioral regulation<br>Beliefs about consequences<br>Knowledge<br>Skills<br>Social influences	Education<br>Modeling<br>Persuasion<br>Training	Demonstration of the behavior<br>Feedback on behavior<br>Information about health consequences<br>Information about social environmental consequences<br>Instruction on how to perform the behavior	Service provision<br>Guidelines<br>Communication/ marketing
14	Document, in patients records, clinical diagnosis (including symptoms) if prescribing an immediate or back up antimicrobial and/or giving self-care advice.	6	UK Department of Health and Public Health England Antimicrobial Prescribing and Stewardship Competencies; NICE Antimicrobial stewardship: systems and processes for effective antimicrobial medicine use [NG15]; TARGET Antibiotics Toolkit; NICE Antimicrobial Stewardship [QS121]; Managing Acute Respiratory Tract Infections (MARTI) e-learning; CENTOR.	Psychological capability	Behavioral regulation<br>Knowledge<br>Skills	Education<br>Enablement<br>Training	Instruction on how to perform the behavior<br>Self-monitoring of behavior<br>Self-monitoring of outcomes of behavior	Service provision<br>Guidelines
15	Undertake POCT in patients 18–65 years old presenting with acute cough/bronchitis in whom antibiotics are being considered.	5	NICE Antimicrobial stewardship: systems and processes for effective antimicrobial medicine use [NG15]; TARGET Antibiotics Toolkit; UK Five Year Antimicrobial Resistance Strategy 2013 to 2018; Public health England Managing Common Infections Guidance; CENTOR.	Psychological capability	Behavioral regulation<br>Knowledge<br>Skills	Training	Instruction on how to perform the behavior	Service provision<br>Guidelines
16	Limit prescribing over the telephone to exceptional cases for self-limiting RTIs.	3	NICE Infection Prevention and Control [QS61]; Center for Pharmacy Postgraduate Education distance course: Antibacterial resistance - a global threat to public health: the role of the pharmacy team; Public health England Managing Common Infections Guidance.	Psychological capability	Knowledge<br>Skills	Training	Instruction on how to perform the behavior	Guidelines
	**Community pharmacists and pharmacy staff (n=5)**							
17	Provide self-care advice for patients with symptoms of self-limiting RTIs, instead of, following or prior to referral to a primary care clinician, giving safety netting advice where appropriate.	7	Public Health England Antibiotic Guardian; TARGET Antibiotics Toolkit; NICE Antimicrobial stewardship: changing risk-related behaviors in the general population [NG63]; Center for Pharmacy Postgraduate Education distance course: Antibacterial resistance - a global threat to public health: the role of the pharmacy team; theLearningpharmacy.com; Royal Pharmaceutical Society: Antimicrobial Stewardship Quick Reference Guide; British Society for Antimicrobial Chemotherapy: Antibiotic Action.	Psychological capability<br>Physical opportunity<br>Reflective motivation	Beliefs about capabilities<br>Environmental context and resources<br>Intention<br>Knowledge<br>Skills<br>Social/professional role and identity	Enablement<br>Persuasion<br>Training	Adding objects to the environment<br>Behavioral practice/rehearsal<br>Commitment<br>Focus on past success<br>Identification of self as role model<br>Instruction on how to perform the behavior	Service provision<br>Guidelines<br>Communication/ marketing
18	Use/share written resources with the public when providing self-care advice for self-limiting RTIs.	5	Public Health England Antibiotic Guardian; TARGET Antibiotics Toolkit; NICE Antimicrobial stewardship: changing risk-related behaviors in the general population [NG63]; Center for Pharmacy Postgraduate Education distance course: Antibacterial resistance - a global threat to public health: the role of the pharmacy team; theLearningpharmacy.com.	Psychological capability<br>Physical opportunity<br>Reflective motivation	Environmental context and resources<br>Intention<br>Knowledge<br>Skills	Enablement<br>Training	Adding objects to the environment<br>Behavioral practice/rehearsal<br>Commitment<br>Instruction on how to perform the behavior	Service provision<br>Guidelines<br>Communication/ marketing
19	When giving an antibiotic prescription for a self-limiting RTI, inform the patients of the dose and duration or to take their antibiotics exactly as prescribed.	3	Public Health England Antibiotic Guardian; Center for Pharmacy Postgraduate Education distance course: Antibacterial resistance - a global threat to public health: the role of the pharmacy team; Royal Pharmaceutical Society: Antimicrobial Stewardship Quick Reference Guide.	Psychological capability<br>Physical opportunity	Environmental context and resources<br>Intention<br>Knowledge	Enablement<br>Training	Adding objects to the environment<br>Behavioral practice/rehearsal<br>Commitment	Service provision<br>Guidelines

				Reflective motivation	Skills		Instruction on how to perform the behavior	
20	Check that antibiotic prescriptions comply with local guidance and query with the prescriber for those that do not.	5	Public Health England Antibiotic Guardian; NICE Antimicrobial stewardship: systems and processes for effective antimicrobial medicine use [NG15]; Center for Pharmacy Postgraduate Education distance course: Antibacterial resistance - a global threat to public health: the role of the pharmacy team; theLearningpharmacy.com; Royal Pharmaceutical Society: Antimicrobial Stewardship Quick Reference Guide.	Psychological capability<br>Physical opportunity<br>Reflective motivation	Environmental context and resources<br>Intention<br>Skills	Enablement<br>Training	Adding objects to the environment<br>Behavioral practice/rehearsal<br>Commitment<br>Instruction on how to perform the behavior	Service provision<br>Guidelines
21	Accept and dispose appropriately of returned antibiotics.	2	Public Health England Antibiotic Guardian; Center for Pharmacy Postgraduate Education distance course: Antibacterial resistance - a global threat to public health: the role of the pharmacy team.	Psychological capability<br>Reflective motivation	Intention<br>Skills	Enablement<br>Training	Behavioral practice/rehearsal<br>Commitment<br>Instruction on how to perform the behavior	Service provision<br>Guidelines
	**Providers and commissioners (n=11)**							
22	Provide education and training in prudent antimicrobial use/AMR (using the antimicrobial resistance and stewardship competencies as a framework).	6	UK Department of Health and Public Health England Antimicrobial Prescribing and Stewardship Competencies; The Health and Social Care Act (HSCA) 2008. Code of Practice on the prevention and control of infections and related guidance; NICE Antimicrobial stewardship: systems and processes for effective antimicrobial medicine use [NG15]; NICE Infection Prevention and Control [QS61]; TARGET Antibiotics Toolkit; UK Five Year Antimicrobial Resistance Strategy 2013 to 2018.	Psychological capability<br>Physical opportunity	Behavioral regulation<br>Environmental context and resources<br>Knowledge<br>Skills	Enablement<br>Training	Action planning<br>Adding objects to the environment<br>Instruction on how to perform the behavior	Service provision<br>Guidelines<br>Legislation
23	Commission, develop or implement interventions (e.g., guidance, services, programs, or campaigns) to support AMS/tackle AMR.	11	Public Health England Antibiotic Guardian; The Health and Social Care Act (HSCA) 2008. Code of Practice on the prevention and control of infections and related guidance; NICE Antimicrobial stewardship: systems and processes for effective antimicrobial medicine use [NG15]; NICE Infection Prevention and Control [QS61]; NHS England Patient Safety Alert – addressing antimicrobial resistance through implementation of an antimicrobial stewardship program; TARGET Antibiotics Toolkit; UK Five Year Antimicrobial Resistance Strategy 2013 to 2018; NICE Antimicrobial stewardship: changing risk-related behaviors in the general population [NG63]; NICE Antimicrobial Stewardship [QS121]; Public Health England Keep Antibiotics Working campaign; Royal College of Nursing (RCN) and Infection Prevention Society (IPS) Infection Prevention and Control Commissioning Toolkit.	Psychological capability<br>Physical opportunity<br>Social opportunity<br>Reflective motivation	Behavioral regulation<br>Beliefs about consequences<br>Environmental context and resources<br>Intention<br>Knowledge<br>Skills<br>Social influences	Education<br>Enablement<br>Incentivization<br>Persuasion<br>Training	Action planning<br>Adding objects to the environment<br>Credible source<br>Feedback on outcome(s) of the behavior<br>Information about health consequences<br>Information about social environmental consequences<br>Instruction on how to perform the behavior<br>Self-monitoring of behavior<br>Social reward<br>Social support (practical)	Service provision<br>Guidelines<br>Communication/ marketing<br>Legislation
24	Commission, develop or implement interventions (e.g., guidance, services, programs, or campaigns) to support self-care.	6	Public Health England Antibiotic Guardian; TARGET Antibiotics Toolkit; PrescQIPP Antimicrobial Stewardship; NICE Antimicrobial stewardship: changing risk-related behaviors in the general population [NG63]; NICE Antimicrobial Stewardship [QS121]; Self Care Forum: Self Care Week.	Psychological capability<br>Physical opportunity<br>Reflective motivation	Behavioral regulation<br>Beliefs about consequences<br>Environmental context and resources<br>Intention<br>Knowledge	Enablement<br>Persuasion<br>Training	Action planning<br>Adding objects to the environment<br>Commitment<br>Information about social environmental consequences<br>Instruction on how to perform the behavior	Service provision<br>Guidelines<br>Communication/ marketing

					Skills		Pros and cons	
25	Monitor antibiotic prescribing in relation to local and national resistance patterns or targets.	12	Public Health England Antibiotic Guardian; UK Department of Health and Public Health England Antimicrobial Prescribing and Stewardship Competencies; The Health and Social Care Act (HSCA) 2008. Code of Practice on the prevention and control of infections and related guidance; NICE Antimicrobial stewardship: systems and processes for effective antimicrobial medicine use [NG15]; NICE Infection Prevention and Control [QS61]; NHS England Quality Premium: 2016/17 Guidance for CCGs; TARGET Antibiotics Toolkit; Public Health England Fingertips platform; PrescQIPP Antimicrobial Stewardship; NICE Antimicrobial Stewardship [QS121]; OpenPrescribing.net; Royal College of Nursing (RCN) and Infection Prevention Society (IPS) Infection Prevention and Control Commissioning Toolkit.	Psychological capability<br>Physical opportunity<br>Social opportunity<br>Reflective motivation<br>Automatic motivation	Behavioral regulation<br>Environmental context and resources<br>Intention<br>Knowledge<br>Reinforcement<br>Social influences	Education<br>Enablement<br>Environmental restructuring<br>Incentivization<br>Training	Adding objects to the environment<br>Commitment<br>Feedback on the behavior<br>Feedback on outcome(s) of the behavior<br>Instruction on how to perform the behavior<br>Material reward<br>Restructuring the physical environment<br>Self-monitoring of behavior<br>Social comparison	Service provision<br>Guidelines<br>Fiscal measures<br>Legislation
26	Promote current national guidelines, or promote/develop local guidelines on antimicrobial prescribing among all prescribers, providing updates if the guidelines change.	6	Public Health England Antibiotic Guardian; The Health and Social Care Act (HSCA) 2008. Code of Practice on the prevention and control of infections and related guidance; NICE Antimicrobial stewardship: systems and processes for effective antimicrobial medicine use [NG15]; NICE Infection Prevention and Control [QS61]; TARGET Antibiotics Toolkit; NICE Antimicrobial stewardship: changing risk-related behaviors in the general population [NG63].	Psychological capability<br>Reflective motivation	Intention<br>Knowledge<br>Skills	Enablement<br>Training	Commitment<br>Instruction on how to perform the behavior	Service provision<br>Guidelines<br>Legislation
27	Provide regular feedback on antimicrobial prescribing and resistance indicators at prescriber, team and organization level benchmarked against local or national antimicrobial prescribing/resistance rates.	5	The Health and Social Care Act (HSCA) 2008. Code of Practice on the prevention and control of infections and related guidance; NICE Antimicrobial stewardship: systems and processes for effective antimicrobial medicine use [NG15]; NICE Infection Prevention and Control [QS61]; TARGET Antibiotics Toolkit; NICE Antimicrobial Stewardship [QS121].	Psychological capability	Behavioral regulation<br>Knowledge<br>Skills	Education<br>Training	Feedback on the behavior<br>Instruction on how to perform the behavior<br>Self-monitoring of behavior	Service provision<br>Guidelines<br>Legislation
28	Provide feedback to prescribers on patient safety incidents related to antimicrobial use, including hospital admissions for potentially avoidable life-threatening infections, infections with Clostridium difficile or adverse drug reactions, such as anaphylaxis.	1	NICE Antimicrobial stewardship: systems and processes for effective antimicrobial medicine use [NG15].	Psychological capability	Behavioral regulation<br>Skills	Education<br>Training	Instruction on how to perform the behavior<br>Self-monitoring of outcomes of behavior	Guidelines
29	Providers have a formulary in place for antibiotic prescribing. *	5	The Health and Social Care Act (HSCA) 2008. Code of Practice on the prevention and control of infections and related guidance; NICE Antimicrobial stewardship: systems and processes for effective antimicrobial medicine use [NG15]; NICE Infection Prevention and Control [QS61]; TARGET Antibiotics Toolkit; Royal College of Nursing (RCN) and Infection Prevention Society (IPS) Infection Prevention and Control Commissioning Toolkit.	Psychological capability	Knowledge<br>Skills	Training	Instruction on how to perform the behavior	Service provision<br>Guidelines<br>Legislation
30	Commissioners seek evidence/providers make evidence available for adherence to local or national guidance for antibiotic prescribing.**	5	NICE Infection Prevention and Control [QS61]; TARGET Antibiotics Toolkit; UK Five Year Antimicrobial Resistance Strategy 2013 to 2018; NICE Antimicrobial Stewardship [QS121]; Royal College of Nursing (RCN) and Infection Prevention Society (IPS) Infection Prevention and Control Commissioning Toolkit.	Psychological capability<br>Physical opportunity	Behavioral regulation<br>Environmental context and resources<br>Knowledge<br>Skills	Education<br>Enablement<br>Environmental restructuring<br>Training	Action planning<br>Instruction on how to perform the behavior<br>Restructuring the physical environment<br>Self-monitoring of behavior	Service provision<br>Guidelines

31	Commissioners ensure information and resources are available for healthcare professionals to use during consultations with people seeking advice about managing self-limiting RTIs. **	2	TARGET Antibiotics Toolkit; NICE Antimicrobial stewardship: changing risk-related behaviors in the general population [NG63].	Psychological capability	Knowledge	Training	Instruction on how to perform the behavior	Service provision<br>Guidelines
32	Reduce antibiotic prescribing/antimicrobial resistance – general behaviors.	7	The Health and Social Care Act (HSCA) 2008. Code of Practice on the prevention and control of infections and related guidance; NHS England Quality Premium: 2016/17 Guidance for CCGs; TARGET Antibiotics Toolkit; Public Health England Fingertips platform; NICE Antimicrobial stewardship: changing risk-related behaviors in the general population [NG63]; NICE Antimicrobial Stewardship [QS121]; British Society for Antimicrobial Chemotherapy: Antibiotic Action.	Psychological capability<br>Physical opportunity<br>Reflective motivation	Behavioral regulation<br>Environmental context and resources<br>Goals<br>Knowledge<br>Skills<br>Social/professional role and identity	Education<br>Enablement<br>Persuasion<br>Training	Action planning<br>Adding objects to the environment<br>Feedback on the behavior<br>Feedback on outcome(s) of the behavior<br>Goal-setting behavior<br>Identification of self as role model<br>Information about health consequences<br>Instruction on how to perform the behavior<br>Self-monitoring of behavior	Service provision<br>Guidelines<br>Legislation

* Providers only ** Commissioners only.
